# Calendula glycolic extract enhances wound healing of alginate hydrogel

**DOI:** 10.1590/acb399724

**Published:** 2024-11-29

**Authors:** Gisele de Oliveira Krubniki Possa, Solange Chopek, Airton Vicente Pereira, Adriana Yuriko Koga, Marcia Regina Paes de Oliveira, Michele Dietrich Moura Costa

**Affiliations:** 1 Universidade Estadual de Ponta Grossa – Postgraduate Program in Biomedical Sciences – Ponta Grossa (PR) – Brazil.; 2 Universidade Estadual de Ponta Grossa – Department of Pharmaceutical Sciences – Ponta Grossa (PR) – Brazil.; 3 Universidade Estadual de Ponta Grossa – Department of Medicine – Ponta Grossa (PR) – Brazil.; 4 Universidade Estadual de Ponta Grossa – Department of Structural, Molecular and Genetic Biology – Ponta Grossa (PR) – Brazil.

**Keywords:** Biopolymers, Wound Healing, Hydrogels, Alginates, Calendula

## Abstract

**Purpose:**

To assess the cytotoxicity and wound healing properties of an alginate hydrogel containing calendula glycolic extract.

**Methods:**

Cell viability in murine fibroblasts (3T3 cells) was evaluated using MTT and SRB assays. The wound healing effect was tested in an incisional wound model on 50 female Wistar rats, divided into two groups: rats treated with alginate hydrogel (n = 25), and rats treated with calendula-alginate hydrogel. Wound healing was assessed by measuring wound retraction rate and histological analysis of lesion tissues over a 28-day period. Histological analyses were performed on days 2, 7, 14, 21, and 28 post-surgery to examine inflammatory infiltrate, macrophage count, and angiogenesis. Picrosirius red staining was used to compare the relative abundance of collagen types I and III fibers.

**Results:**

Cytotoxicity tests on 3T3 cells revealed increased cell viability with the calendula-alginate hydrogel. The calendula-alginate hydrogel also demonstrated a significant improvement in wound closure, supported by histopathological analysis, showing reduced inflammation, increased macrophage activity, and enhanced collagen deposition.

**Conclusions:**

These findings evidenced the therapeutic potential of combining calendula extract and alginate for promoting enhanced wound healing.

## Introduction

For centuries, medicinal plants and their preparations have been extensively used in traditional medicine for the treatment of wounds. Nowadays, approximately 40% of medicines are derived from natural sources and traditional knowledge. Additionally, many modern synthetic drugs are developed based on the chemical structures of compounds isolated from plants^1^. In this context, various formulations containing herbal extracts, and their isolated metabolites have been developed and tested in both in-vitro and animal studies to assess their effectiveness in wound healing^2^.

Alginate, a natural polysaccharide derived from brown seaweed, is widely known for its water-soluble and gel-forming properties. Alginate-based hydrogels are commonly used in wound care due to their non-toxic, biocompatible, and biodegradable nature, as well as their ability to retain moisture, which helps create a natural microenvironment for the skin^3^.

Several hydrogels with enhanced wound healing properties have been proposed by incorporating medicinal plant extracts. This therapeutic approach for wound care has been demonstrated in hydrogels containing herbal extracts such as *Aloe vera* 4 ^,^ 5, *Raphanus sativus* 6, and Amaranthus spinosus-Rubia cordifolia^7^.

*Calendula officinalis* Linn., commonly known as pot marigold or calendula, has been extensively studied for its potential therapeutic benefits in wound healing^8^. Calendula extracts contain bioactive compounds such as glycosylated flavonoids, terpenoids, carotenoids, phenolic acids, tannins, amino acids, and polysaccharides, which exhibit anti-inflammatory and antimicrobial properties, promoting the healing of minor skin lesions and wounds^9^.

The hydroalcoholic extract of calendula in a carbopol gel has shown remarkable results in promoting wound healing in albino mice^10^. It effectively stimulated collagen synthesis, increased granuloma tissue, and significantly enhanced wound contraction. However, while calendula glycolic extract is well-known for its dermatological applications, its potential for wound healing has not been thoroughly explored. A study on a 4% calendula glycolic extract topical cream for Achilles tendon lesions demonstrated the extract’s effectiveness in enhancing wound healing^11^. Additionally, incorporating polyols such as glycerol into the hydrogel matrix can absorb excess wound exudate. Glycerol helps prevent exudate accumulation, creating an optimal environment for effective wound healing^12^.

In this study, a hydrogel combining calendula glycolic extract and alginate was used for the first time to evaluate its wound healing effects. This novel alginate hydrogel formulation was prepared and subsequently tested *in vitro* (MTT and SRB assays) and *in vivo* (dorsal incisional wound model in rats). The results were compared to those obtained with an alginate hydrogel without calendula extract.

## Methods

### Plant material

Dried whole flowers of *Calendula officinalis* Linn, specifically ligulate flowers, were obtained from Chamel, Paraná, Brazil. Macroscopic and microscopic characteristics of the species were previously confirmed, and the total flavonoid content (TFC) in calendula powder was determined as recommended in the sixth edition of Brazilian Pharmacopoeia^13^.

### Glycolic calendula extract preparation

Ten grams of finely powdered calendula flower petals (250–500 microns) were macerated in 100 mL of a polyethylene glycol-water (9:1) solution in an amber glass flask at room temperature for 14 days with intermittent stirring. The extract was then vacuum filtered, collected, and kept in an amber flask.

### Calendula-alginate hydrogel preparation

Alginate solution at 1.5% (m/v) was prepared by adding 1.5 g of sodium alginate to 100 mL of deionized water. The mixture was heated in a water bath at 40°C until a viscous solution formed, after which 1 g of sodium CMC was added and dispersed using an overhead stirrer at 500 rpm for 15 min. Finally, the calendula glycolic extract (10% v/v) was incorporated into the alginate hydrogel and stirred for 5 min to achieve a homogeneous mixture. The hydrogel was sterilized under a germicidal lamp (Philips TUV 15W/G15 T8) in a laminar flow hood (Veco CFLH-09) for 15 min.

### Calendula-alginate hydrogel characterization

Before the use in cytotoxicity and animal assays, the alginate-calendula hydrogel underwent various physicochemical tests. These tests included stability by centrifugation, pH measurements, and viscosity analysis. For the centrifugation test, 10 g of the hydrogel was placed in a centrifuge tube and centrifuged at 3,000 rpm for 3 min at 20°C using a Parsec CT-0603-IC centrifuge. The pH was determined using a pre-calibrated digital pH meter (Digimed DMPH-2) with pH 4 and 7 buffer solutions. Viscosity was measured at 20°C using a Brookfield viscometer (Quimis Q860M21) with spindle number 4 at 6 rpm, and the results were expressed in Pascal.seconds (Pa.s).

### Total flavonoid content

The TFC in glycolic extract was determined spectrophotometrically based on the reaction of flavonoids with AlCl3 and measuring absorbance at 425 nm. The TFC was calculated using a quercetin analytical curve in the range of 2.5 to 20 µg·mL-^1^.

### In-vitro cytotoxicity

In-vitro cytotoxicity tests were performed through MTT and SRB assays for the solvent mixture, alginate hydrogel, and calendula-alginate hydrogel against the murine fibroblast 3T3 cell line. Briefly, cells were cultured in Dulbecco’s modified eagle medium (DMEM, low glucose) supplemented with 10% fetal bovine serum (FBS), 100 U/mL penicillin, and 100 µg/mL streptomycin (Gibco), along with HEPES buffer and sodium bicarbonate (Sigma). Cells were incubated in a CO2 incubator at 37°C with a humidified atmosphere of 5% CO_2_ and subcultured twice a week. Fibroblast were then seeded in 96-well plates and treated with various sample dilutions for 48 hours.

### Wound healing assay

The assay was approved by the Ethics Committee on Animal Experimentation (registry number 004885). Fifty female Wistar rats (weight 250–280 g) were divided into two groups (n = 25):

- Alginate hydrogel (control);
- Calendula-alginate hydrogel.

Anesthesia was administered via intraperitoneal injections of ketamine (40 mg/kg) and xylazine (5 mg/kg). A 2 × 2 cm cutaneous wound was surgically induced on the dorsum under aseptic conditions using a scalpel. The animals were housed individually and provided with *ad libitum* access to food and water. One gram of hydrogel was applied daily to the lesions for seven days. On postoperative days 2, 7, 14, 21, and 28, five rats (n = 5) from each group were euthanized with an overdose of isoflurane, and the wounded areas were surgically removed for histological analysis.

### Wound area measurements

The areas of the wounds of each animal (n = 5) were measured on days 2, 7, 14, 21, and 28 using Image J software. The results were expressed as the percentage of wound retraction of the original wound area (mm^2^).

### Histological analysis

Samples collected on days 2, 7, 14, 21, and 28 were fixed in 3.7% (v/v) buffered formaldehyde at room temperature for 24 hours followed by paraffin embedding. Five-micrometer thick sections were prepared and stained with hematoxylin and eosin (HE) to evaluate the presence of inflammatory infiltrates, macrophages presence, and angiogenesis. The HE-stained samples were examined under a light microscope (Olympus DP72), and images were captured using cellSens Standard software. Inflammatory cells and blood vessels were quantified using ImageJ’s Cell Counter plugin.

### Picrosirius red

On days 14, 21, and 28, collagen content was assessed using picrosirius red-stained sections. Collagen fibers were visualized under crossed-polarized light with a polarization microscope (Olympus LG-PS2). Birefringence patterns were analyzed, and collagen content was quantified using ImageJ software. A threshold was set to distinguish type I collagen (red) from type III collagen (green). The total collagen area was then calculated and expressed in pixels.

### Statistical analysis

Data from the in-vitro assays were analyzed using the GraphPad Prism software (version 7) and expressed by mean ± standard deviation, with the significance level of 5% (*p* ≤ 0.05). Analysis of variance (ANOVA) followed by Tukey’s post-test was used to analyze and compare the means of the treatments. Data from the *in-vivo* experiments were analyzed using IBM Statistical Package for the Social Sciences (version 2.1). ANOVA followed by Tukey’s post-test was also applied to compare the results.

## Results

### Physicochemical properties of calendula-alginate hydrogel

**Figure 1:**
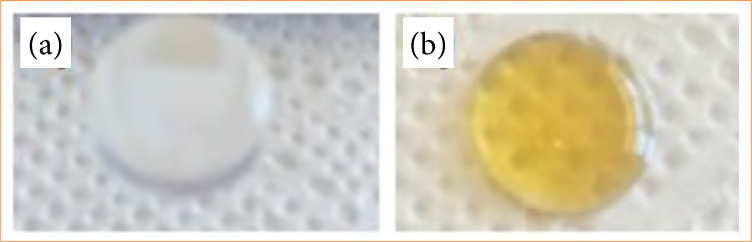
Digital images of the formulations **(a)** alginate hydrogel and **(b)** calendula-alginate hydrogel.

Source: Elaborated by the authors.

The flowers of *Calendula officinalis* are typically yellow or orange, primarily due to the presence of carotenoids. These carotenoids are effectively extracted using glycolic solvents such as glycerol and propylene glycol. The incorporation of calendula glycolic extract into the alginate hydrogel formulation imparted the typical orange color of calendula flowers to the hydrogel (Fig. 1).

The stability of the hydrogel formulations was evaluated by assessing their physicochemical properties, including phase separation, pH, and viscosity. Centrifugation tests revealed no evidence of precipitation or phase separation in the calendula-alginate hydrogel formulation.

The pH of the alginate-CMC hydrogel was 6.5, while the calendula glycolic extract had a slightly more acidic pH of 5.8. The incorporation of the glycolic calendula extract (10% v/v) into the alginate hydrogel resulted in a final pH of 5.9.

Sodium carboxymethylcellulose (CMC), a water-soluble polysaccharide, was used as a viscosity modifier. Incorporating the calendula glycolic extract into the alginate-CMC hydrogel caused a slight reduction in viscosity from 25.1 ± 0.3 to 21.3 ± 0.5 Pa.s.

### Total flavonoids content in the calendula flowers and glycolic extract

According to the Brazilian Pharmacopoeia^13^, calendula flowers should contain at least 0.40% of total flavonoids. In this study, the TFC of the dried and crushed calendula flowers was 0.76%, expressed as hyperoside (C_21_H_20_O_12_). For the glycolic extract, the TFC, expressed as quercetin, was found to be 1.66% (m/v).

### Cell viability

**Figure 2:**
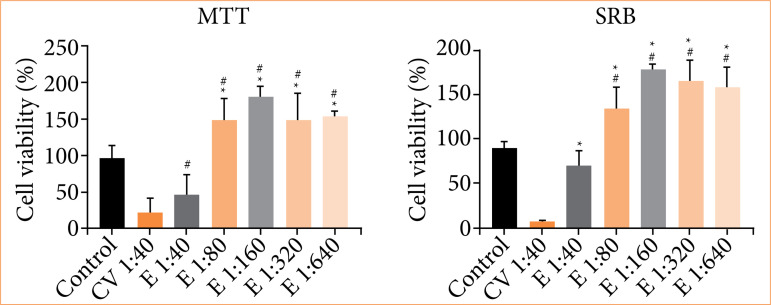
Cell viability of 3T3 cells by MTT and SRB assays after 48 hours. Conditions: Dulbecco’s modified eagle medium (control), vehicle control (VC), and calendula extract **(E)** at dilutions in the range of 1:40 to 1:640.

The results of MTT and SRB assays indicated that glycolic calendula extract (Fig. 2) decreased cell viability at a 1:40 dilution after 48 hours of exposure. However, at higher dilutions, the viability of 3T3 cells increased. In contrast, the extractor solution (polyethylene glycol:water [9:1]) induced significant cell death. Overall, both assays demonstrated that calendula glycolic extract could enhance 3T3 cell proliferation at higher dilutions.

The MTT assay of 3T3 cells treated with the calendula-alginate hydrogel (Fig. 3) showed comparable viability to the control group at a 1:40 dilution. Increased cell viability was observed at higher dilutions (1:160 to 1:640). The vehicle control also exhibited cell viability comparable to the DMEM control.

#significant difference (*p* > 0.05) from the control; *significant difference (*p* > 0.05) from the vehicle. Source: Elaborated by the authors.

**Figure 3:**
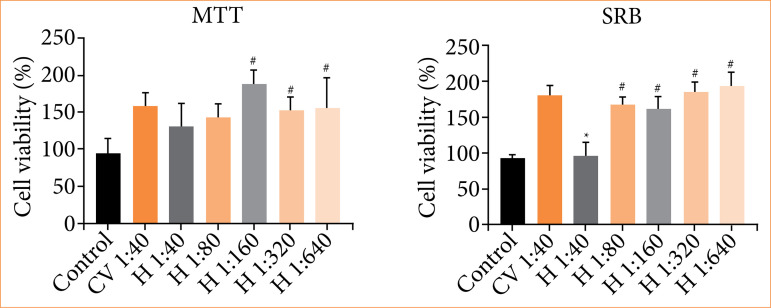
Cell viability of 3T3 cells treated with calendula-alginate hydrogel using MTT and SRB assays after 48 hours. Conditions: Dulbecco’s modified eagle medium (control), vehicle control (VC), and calendula-alginate hydrogel **(H)** at dilutions ranging from 1:40 to 1:640.

#significant difference (*p* > 0.05) from the control; *significant difference (*p* > 0.05) from the vehicle. Source: Elaborated by the authors.

SRB assay results (Fig. 3) showed similar findings. At a 1:40 dilution, the calendula-alginate hydrogel exhibited cell viability comparable to the control. Higher dilutions (1:80 to 1:640) resulted in significantly increased cell viability. The vehicle control showed comparable cell viability to the calendula-alginate hydrogel treatments.

### Wound healing assay

#### Wound retraction

**Figure 4:**
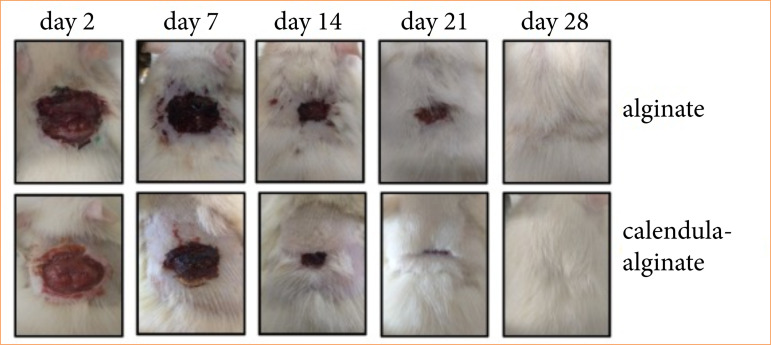
Macroscopic images of wounds treated with alginate hydrogel (control) and calendula-alginate hydrogel on days 2, 7, 14, 21, and 28 post-surgery.

Source: Elaborated by the authors.

**Figure 5:**
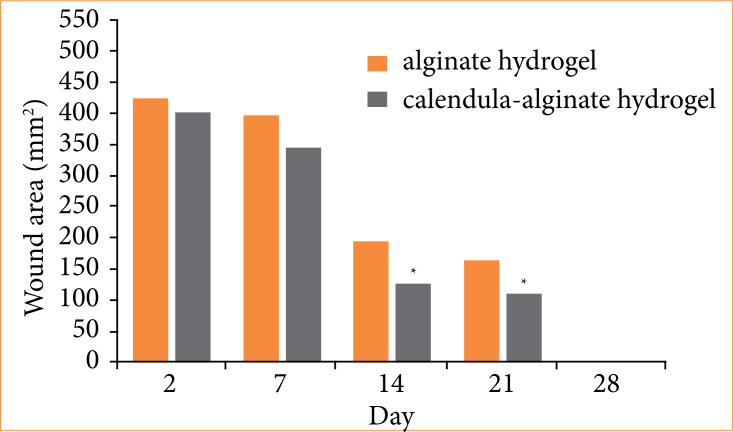
Wound retraction following treatment with alginate hydrogel (control) and calendula-alginate hydrogel.

Source: Elaborated by the authors.

Wound retraction was evaluated by measuring the wound area on postoperative days 2, 7, 14, 21, and 28 (Fig. 4). Significant differences in wound retraction were observed between the calendula-alginate hydrogel and control groups on the 14th and 21st days (*p* = 0.005 and *p* = 0.05, respectively) (Fig. 5).

### Histological assays

**Figure 6:**
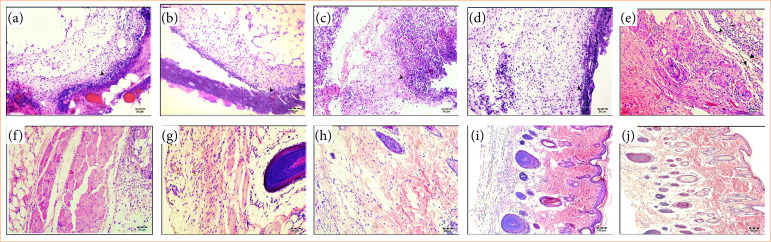
Photomicrographs of the tissue wounds stained with hematoxylin-eosin treated with alginate hydrogel at the **(a)** second; **(c)** seventh; **(e)** 14th; **(g)** 21st and **(i)** 28th days; and calendula-alginate hydrogel at the **(b)** second; **(d)** seventh; **(f)** 14th; **(h)** 21st; and **(j)** 28th days.

**Figure 7:**
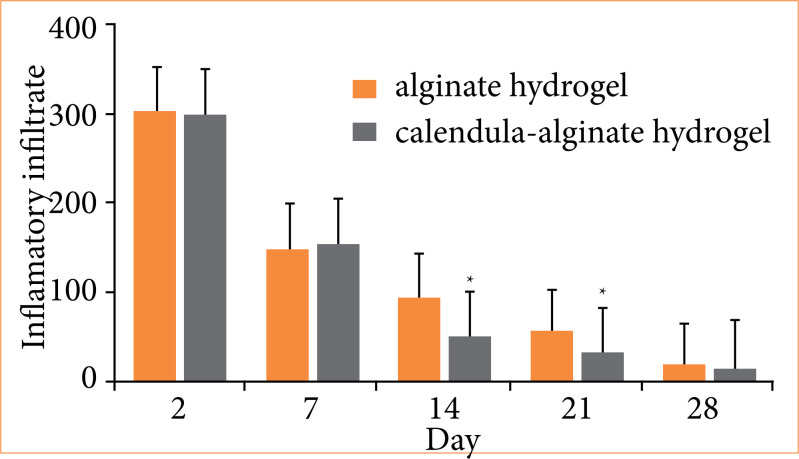
Inflammatory infiltrate in wounds treated with alginate hydrogel (control) and calendula-alginate hydrogel on the second, seventh, 14th, 21st, and 28th days.

The wound healing process was evaluated histologically over 28 days. By the 28th day, both treatments showed signs of complete healing (Fig. 6). Histological analysis revealed an acute inflammatory response in both groups on the second and seventh days. By the 14th and 21st days, the calendula-alginate hydrogel group showed significantly reduced inflammatory infiltrate compared to the control group (*p* < 0.001) (Fig. 7).

⬜: inflammatory infiltrate; →: macrophages; •: blood vessels. Source: Elaborated by the authors.

*p ≤ 0.05. Source: Elaborated by the authors.

**Figure 8:**
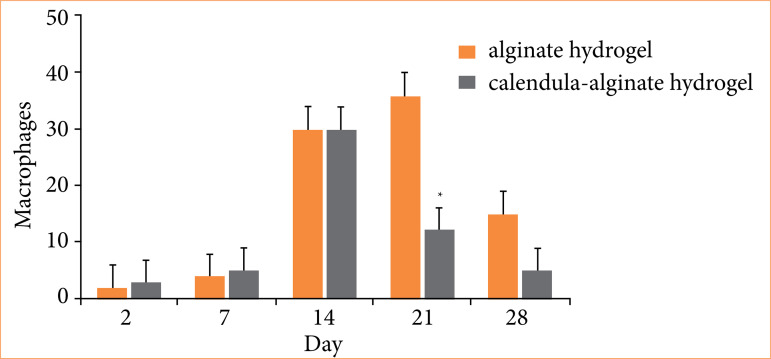
Macrophages in the wounds treated with alginate hydrogel (control) and calendula-alginate hydrogel on the second, seventh, 14th, 21st, and 28th days.

**Figure 9:**
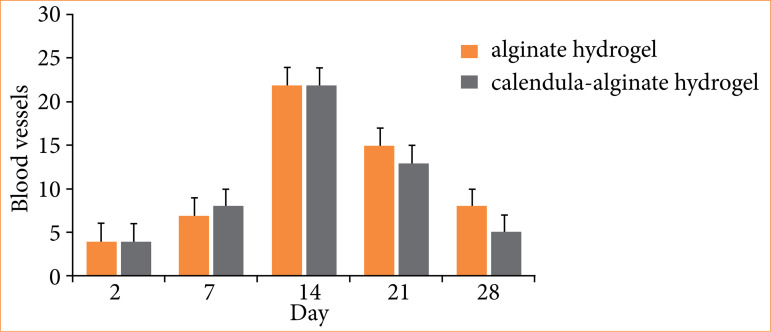
Blood vessels in the wounds treated with alginate hydrogel (control) and calendula-alginate hydrogel on the second, seventh, 14th, 21st, and 28th days.

Quantitative histological analysis revealed a significantly higher macrophage count in the control group on the 21st day compared to the calendula-alginate hydrogel group (*p* = 0.004) (Fig. 8). No significant differences in angiogenesis were observed between the groups (Fig. 9).

*p ≤ 0.05. Source: Elaborated by the authors.

*p ≤ 0.05. Source: Elaborated by the authors.

**Figure 10:**
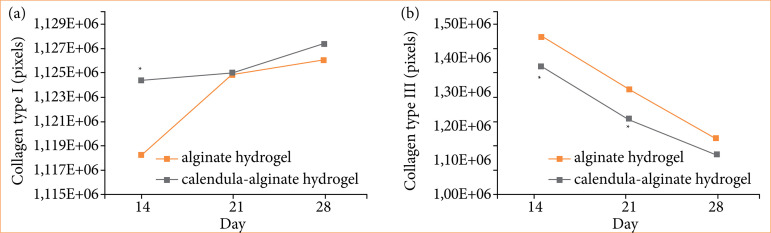
Comparative analysis of collagen types in wound healing. **(a)** Collagen type I and **(b)** type III in the groups treated with alginate hydrogel (control) and calendula-alginate hydrogel on days 14, 21, and 28.

Source: Elaborated by the authors.

During wound maturation, type III collagen is gradually replaced by type I collagen. Picrosirius red staining was used to quantify collagen types in wound tissues. By day 14, a significant increase in type I collagen was observed in the calendula-alginate hydrogel group compared to the control. No significant differences were observed in type I collagen levels on the 21st and 28th days. Additionally, a significant decrease in type III collagen was observed in the calendula-alginate group on days 14 (*p* = 0.03) and 21 (*p* = 0.01) compared to the control (Fig. 10).

## Discussion

Our study aimed to evaluate the efficacy of calendula bioactive compounds in enhancing wound healing when incorporated into an alginate hydrogel, and the results have been quite promising. The flavonoid content of the calendula flowers used in this study was quantified as hyperoside, with a concentration of 0.76%, exceeding the minimum requirement of 0.40% set by the Brazilian Pharmacopoeia^13^. This higher concentration possibly contributes to the observed improvement in wound healing, as flavonoids are known for their antioxidant and anti-inflammatory properties, which are essential in the wound repair process.

The incorporation of calendula glycolic extract to the alginate hydrogel formulation resulted in a pH reduction from 6.5 to 5.9, which corresponds closely with the natural pH of the skin. Studies have shown that slight acidity is conducive to wound healing, especially in chronic wounds. The wounds progressing towards healing often exhibit a pH shift, moving from acidic to neutral and back to acidic as epithelialization occurs^14^. The pH adjustment induced by the glycolic calendula extract likely contributed to more effective wound closure observed, particularly in the early stages of wound healing.

Notably, the addition of glycolic extract, prepared by the maceration of calendula flowers with propylene glycol-water (9:1), altered the viscosity of the alginate hydrogel, suggesting an interaction between the extract and the hydrogel matrix. While methanol and ethanol are commonly used as solvents to extract calendula compounds, methanol is highly toxic to the skin, and compounds extracted with ethanol, such as polyphenols and flavonoids, are poorly soluble in hydrogels due to their high-water content. In contrast, propylene glycol serves as an effective moisturizer, helping to maintain a moist wound environment, which is critical for optimal wound healing^15^. The physicochemical characterization of the calendula-alginate hydrogel emphasizes the importance of considering solvent composition in herbal extract formulations for therapeutic use.

Cytotoxicity assessment is an essential step in evaluating the safety and effectiveness of topical formulations. In our study, the calendula glycolic extract, incorporated at 10% (v/v) in the alginate hydrogel, demonstrated no cytotoxic effects. Instead, it promoted cell proliferation as indicated by both MTT and SRB assays. This suggests that calendula extract does not induce cytotoxicity, but it may support cellular processes essential for wound healing. Calendula contains triterpenes, such as faradiol esters, which likely contribute to wound healing by stimulating fibroblast activity^16^^–^ 18. Although propylene glycol was cytotoxic at high concentrations, its dilution in the calendula-alginate hydrogel mitigated potential harmful effects. Increased cell viability further supports the therapeutic potential of glycolic calendula extract in wound care.

The wound healing assay in rats demonstrated that the calendula-alginate hydrogel significantly accelerated wound retraction by day 14, indicating more effective wound closure compared to the control alginate hydrogel. This effect is likely due to the bioactive calendula compounds, such as flavonoids and triterpenoids, which are known to promote fibroblast and keratinocyte migration and proliferation^19^, fundamental cellular events in wound contraction, re-epithelialization, and reducing the healing time.

Histological analysis showed reduced inflammatory infiltration, evidencing the anti-inflammatory properties of the calendula-alginate gel. The significant decrease in inflammatory cells, especially on days 14 and 21, suggests that active constituents of calendula flowers, such as faradiol monoesters and flavonol glycosides, effectively modulate the inflammatory response, thereby promoting faster wound healing^20^^–^ 22.

However, despite various studies showing the positive effects of calendula on wound healing, our results did not reveal significant differences in angiogenesis. This finding contrasts with previous studies in which calendula extract enhanced blood vessel formation in the chorioallantoic chicken membrane assay^23^. This discrepancy suggests the need to optimize experimental protocols and standardize the concentration of bioactive compounds to better understand their effects on neovascularization during wound healing.

Moreover, our study showed a significant reduction in type III collagen and an increase in type I collagen in the group treated with calendula-alginate hydrogel. This switch from type III to type I collagen, along with improved collagen fiber organization, indicates enhanced wound strength and more mature scar formation. Calendula extracts may inhibit collagen degradation and support the production of more stable collagen fibers, improving the overall quality of the healed tissue^24^^–^ 26.

## Conclusion

Our study provided evidence that the propylene glycol:H_2_O (9:1) extract of calendula contains a significant concentration of flavonoids. The incorporation of calendula glycolic extract into an alginate hydrogel demonstrated excellent cytocompatibility and enhanced wound healing potential. Our findings indicate a potential synergistic effect of calendula extract in wound care; however, further research is needed to better understand the phytochemical composition of calendula glycolic extract and enhance its therapeutic use.

## Data Availability

All data sets were generated or analyzed in the current study.
